# The Influence of Temperature on the Behavior of Burrowing in Larvae of the Blowflies, *Chrysomya albiceps* and *Lucilia cuprina*, Under Controlled Conditions

**DOI:** 10.1673/031.009.1401

**Published:** 2009-04-21

**Authors:** Leonardo Gomes, Guilherme Gomes, Claudio J. Von Zuben

**Affiliations:** ^1^ Departamento de Biologia, Avenida 24A, 1515, UNESP - São Paulo State University - Rio Claro, SP, Brazil; ^2^ Departamento de Zoologia, Avenida 24A, 1515, UNESP - São Paulo State University - Rio Claro, SP, Brazil; ^3^ Bolsista do CNPq

**Keywords:** blowflies, burrowing behavior, larval dispersal, temperature

## Abstract

Blowflies use discrete, ephemeral substrates for larval development. After exhaustion of the food supply, larvae will disperse in search of sites to burrow and pupate or will seek other sources of food in a process known as post-feeding larval dispersal. In this study, the effect of temperature was investigated as it is one of the most important aspects of the environmental variables in this process. 800 larvae of the blowflies *Chrysomya albiceps* (Wiedemann 1819) and *Lucilia cuprina* (Wiedemann 1830) (Diptera: Calliphoridae) were incubated in tubes covered with vermiculite at 15, 20, 25 and 30°C. For each pupa, the body weight, sex and depth of burrowing were determined. Statistical tests were used to examine the relationship of depth of burrowing and body weight to the temperature at which burrowing occurred. Depth of burrowing was affected differently by temperature for both of the species studied; *L. cuprina* larvae burrowed deeper at lower and higher temperatures while *C. albiceps* larvae burrowed less at extreme temperatures. Additionally, temperature had a significant effect on the body weight of *L. cuprina* larvae as body weight decreased as temperature increased, whereas for *C. albiceps*, pupal weight increased up to 25°C and then decreased abruptly at a higher temperatures. The maximum body weight was also differently affected in the two species; in *L. cuprina*, the maximal weight was at 15°C and for *C. albiceps* weight was maximal at 20°C.

## Introduction

Blowflies of the genera *Chrysomya* and *Lucilia* (Diptera: Calliphoridae) are of considerable medical and sanitary importance since they carry enteropathogens, such as viruses, bacteria and helminths (Lima and Luz 1991 ) and may cause myiasis in animals and humans (Zumpt 1965; Guimarães et al. 1983). These flies are also important in forensic entomology since they can be used to determine the post mortem interval (Ullyett 1950; Smith 1986; Greenberg 1990; Gomes et al. 2003, 2005, 2006).

The substrates in which blowflies develop are discrete and ephemeral (Atkinson and Shorrocks 1981; Ives 1989; Ives 1991). As a result, the larval stage is the main period in which blowflies face limited food resources. Each food source is normally heavily colonized by insects of one or more species, and there is often intense competition for resources (Hanski 1987). Once the food resource has become unsuitable or depleted, the larvae leave in search of a place to pupate, or in search of another source of food if they have not achieved the minimal weight required for pupation. The process of abandonment of the food resource is known as post-feeding larval dispersal, which will expose the larvae to a number of constraints, such as abiotic changes, with temperature and photoperiod being considered the most important (Greenberg 1990; Gomes et al. 2002, 2005).

Several laboratory studies have investigated post-feeding larval dispersion in blowflies (Greenberg 1990; Gomes et al. 2002, 2003, 2005, 2006). Although some field studies have also been reported (Greenberg 1990; Tessmer and Meek 1996; Gomes *et al*. 2007), most have suffered from the inability to control environmental variables as easily as in the laboratory. This is a critical consideration since one of the most important questions is how climatic conditions can affect post-feeding larval dispersal and the subsequent burrowing of the larvae prior to pupation.

The study of larvae burying behavior is important to improve understanding of one of the processes during larval dispersion, and to try to understand the influence of environmental variables (in this case, temperature) on this behavior. Furthermore, this could help to determinate the location of larvae buried in the soil around a carcass or a decomposing body for a forensic entomologist, giving more data about the environmental conditions after death until the body is found, and helping to determine the postmortem interval.

Furthermore, the study of burying behavior of *L. cuprina* (Wiedemann 1830) and *C. albiceps* (Wiedemann 1819) is very important because adults of these species are of extreme importance in forensic entomology as they are the first to colonize a dead body during the initial decomposition stages. Consequently, larvae from these species are the first to disperse and bury under or around the body (Gomes et al. 2006).

Thus, to address this question, we examined the burrowing behavior of *C. albiceps* and *L. cuprina* larvae in response to different conditions of temperature by assessing depth of burrowing and larval weight.

## Materials and Methods

*C. albiceps* and *L. cuprina* were field collected from Rio Claro, São Paulo, and maintained in cages (30 × 30 × 30 cm) covered with nylon and were fed water and sugar *ad libitum* under controlled conditions (25 ± 1°C, 70 ± 10% RH, and 12:12 L:D). Adult females were fed fresh beef liver to allow complete development of the gonotrophic cycle (Linhares 1988). Newly hatched larvae of both species were obtained from adult flies kept at 25°C and 70% relative humidity, and were raised in vials containing 50 g of ground beef.

Four hundred of each species in the final third instar that were 6 days old were used for these experiments. All larvae of a given species were similar in weight at the beginning of the experiment, although the mean body weights of the two species differed (25.34 ± 1.7 mg and 33.57 mg ± 1.3 mg for *C. albiceps* and *L. cuprina* larvae, respectively). The larvae (100 for each treatment group) were placed individually in test tubes (30 cm × 2 cm) containing vermiculite and incubated at 60% ± 10 relative humidity on a 12 h photoperiod at 15°C, 20°C, 25°C and 30°C to allow them to burrow and pupate. These conditions, including tube size, were used to ensure that any movement of the larvae was directed towards burrowing. Previous work demonstrated that, regardless of the type of substrate available for pupation, the larvae would not bury themselves deeper than an average of 15 cm (Gomes *et al*. 2005).

After they had pupated, the pupae were located and removed from the vermiculite. The depth of the pupation site was measured. Each pupa was then placed separately in a plastic flask and weighed on an Ohaus® analytical balance (www.ohaus.com). All of pupae located in the vermiculite were weighed at the same time to avoid mistakes in the comparisons of means, and pupal weight was measured for 10 day-old pupae. After weighing, each pupa was returned to its flask for sexing after emergence of the adult.

The results were expressed as the mean and standard deviation (S.D.). The Tukey test was used to compare the means of variables and linear regression was used to determine the relationships among the variables (Zar 1999).

**Figure 1.**
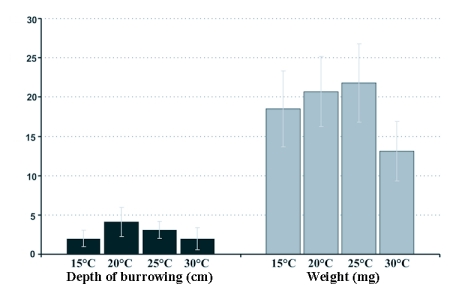
Body weight and depth of burrowing for *C. albiceps* pupae at different temperatures.

## Results and Discussion

**Figure 2.**
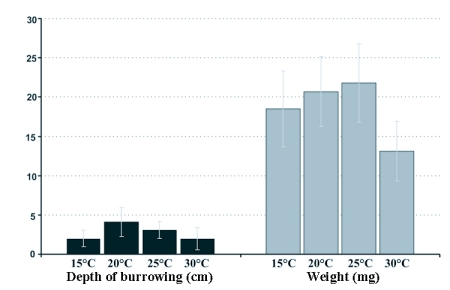
Body weight and depth of burrowing for *L. cuprina* pupae at different temperatures.

Body weight and depth of burrowing were affected for both species of flies studied (Figures 1 and 2), with *L. cuprina* being more sensitive than *C. albiceps* to changes in higher temperatures.

Temperature affected the depth of burrowing (Figures 1 and 2) (F = 19.34, p <0.05). Larvae of *C. albiceps* burrowed themselves deeper at a more amenable temperature (20°C) to pupate, remaining closer to the surface at the lower and higher temperatures used in this study. Pupal weight decreased significantly (F = 18.23, p <0.05) at the extreme temperatures, peaking at 25°C. For *C. albiceps* pupae, there appeared to be an optimal temperature for maximum burrowing (20°C), with less burrowing at less favorable lower and higher temperatures. Weight increased up to 25°C but decreased thereafter. There were significant differences in the mean values of depth of burrowing (F = 21.89, p <0.05) in relation to temperature.

In contrast, for *L. cuprina* pupae, the depth of burrowing did not vary significantly with temperature, but there was a significant progressive decrease in body weight with increasing temperature (F = 5.13, p <0.001).

Linear regression showed that there was a significant relationship between the increase in temperature and the decrease in body weight for *L. cuprina* pupae (Body weight = 23.78 ± 0.20 × Temperature (F = 14.941; p <0.001, r^2^ = 0.76).

Therefore, temperature had significant effects on the burrowed depth or in the pupal weight, depending on species. *L. cuprina* pupa weight was lowest at higher temperatures while *C. albiceps* larvae burrowed less at extreme temperatures. In contrast, in the case of *L. cuprina*, although temperature had no significant effect on the depth of burrowing, there was nevertheless a trend towards shallower burrowing at 20°C. These findings suggested that *C. albiceps* prefers to burrow deeper at a temperature of 20°C, whereas *L. cuprina* prefers to burrow deeper at extreme temperatures. A greater sensitivity of *L. cuprina* larvae to extreme temperatures could have allowed deeper burrowing at low and high temperatures. In addition, some of this variation in depth of burrowing could also be related to the tendency of *L. cuprina* larvae to burrow less deeply than *C. albiceps* larvae under normal conditions.

Temperature had a significant effect on body weight in both species. In *L. cuprina* larvae, there was a progressive decrease in body weight with increasing temperatures, whereas for *C. albiceps* larvae, body weight increased up to 25°C and then decreased abruptly at 30°C.

Assuming that *C. albiceps* larvae feed more when conditions are most favorable, one may infer that the most favorable temperature for this species is between 20°C and 25°C for which the greatest body weight and greatest depth of burrowing were recorded. However, in fed larvae such as those used here, an increase in temperature will increase the rate of metabolism and water loss, and reduce body weight because of additional energy consumption. In the case of *L. cuprina* larvae, the discovery that the greatest body weight was attained below 20°C, and possibly even below 15°C, and that the deepest burrowing occurred at 15°C, indicated that the most favorable temperature for this species was considerably lower than for *C. albiceps*.

Temperature may alter the burrowing behavior of larvae prior to pupation when compared with previous studies in normal conditions in the laboratory (Gomes et al. 2005, 2006). At low temperatures, the metabolic rate may be markedly reduced and this could result in greater body weight and a tendency to burrow deeper in order to escape low temperatures (Grassberger and Reiter 2003).

This study can help to understand how the behavior and physiology of these, and other, species of blowflies are affected by environmental conditions as they search for burrowing sites for pupation (Gomes and Von Zuben 2003, 2004a, b). Investigations of the effect of temperature in intervals less than 5°C, as well as humidity and luminosity, could help to understand burying behavior of this and other fly species. Since the environmental variables that influence burying behavior are related to other biological variables such as predation and parasitism, understanding this behavior could help to locate larvae around a dead body, and consequently help in post mortem investigations.

## Editor's note

The authors have attested to the veracity of the data reported in this paper, and that it fairly discusses the work of others.
